# Evidence for Autoimmunity in the Pathogenesis of COVID-19-Induced Myocarditis

**DOI:** 10.3390/ijms27062694

**Published:** 2026-03-16

**Authors:** Ortal Tuvali, Michael Welt, Clara Benaim, Michael Fassler, Jacob George

**Affiliations:** Heart Center, Kaplan Medical Center, Faculty of Medicine, Hebrew University of Jerusalem, Rehovot 76100, Israel

**Keywords:** myocarditis, COVID-19, autoimmunity, molecular mimicry, interferon-gamma, animal model

## Abstract

Myocarditis has been described following SARS-CoV-2 infection. The mechanisms underlying COVID-19-associated myocarditis remain incompletely understood. Peripheral blood mononuclear cells (PBMCs), IgG fractions, and myocardial biopsy tissue were obtained from a patient with COVID-19 myocarditis. Cellular responses to SARS-CoV-2 spike protein and myocardial tissue extract were assessed in vitro. PBMCs and purified IgG were passively transferred into Rag2/IL2RG-/- mice. Interferon-gamma (IFN-γ) production and cardiac IFN-γ transcript levels were measured. PBMCs from the myocarditis patient proliferated in response to spike protein and myocardial tissue extract, whereas PBMCs from a healthy control did not. PBMCs from the patient secreted higher concentrations of IFN-γ compared with the healthy control. Introduction of patient PBMCs or IgG into Rag2/IL2RG-/- mice resulted in higher cardiac IFN-γ transcript levels compared with control PBMCs or IgG. These findings demonstrate cellular reactivity to SARS-CoV-2 spike protein and myocardial tissue, increased IFN-γ production, and induction of cardiac IFN-γ expression following passive transfer of immune components.

## 1. Introduction

COVID-19 infection has been associated with an increased incidence of myocarditis, with COVID-19 patients having a 15.7-fold higher risk compared with uninfected individuals, and incidence rates rising from pre-pandemic estimates of 1–10 per 100,000 to 150–4000 per 100,000 during the pandemic [1,2]. While myocarditis was also reported following widespread vaccination, a substantial number of cases occurred after SARS-CoV-2 infection. The risk of myocarditis is notably higher after infection than vaccination, with an estimated 40 additional cases per million following SARS-CoV-2 infection versus 2–10 per million following vaccination [3,4,5].

The viral spike (S) protein is a type I transmembrane fusion glycoprotein essential for viral entry and propagation [6]. Despite extensive investigation, the mechanisms underlying COVID-19 myocarditis remain incompletely defined. Proposed mechanisms include direct viral injury, immune-mediated inflammation, microvascular injury, cytokine storm, and molecular mimicry [2,7]. SARS-CoV-2 RNA has been detected in the myocardium in up to 25–50% of autopsy specimens, predominantly in pericytes and sub-endothelium rather than within cardiomyocytes, challenging direct myocardial infection as the sole mechanism [2].

Autoimmune disorders may arise through cross-reactive immune responses, dysregulated cytokine networks, altered self-antigens, and molecular mimicry [6]. Molecular mimicry occurs when shared structural motifs between pathogen-derived and host-derived antigens activate autoreactive T or B lymphocytes in genetically susceptible hosts [6,8].

Recent studies have raised the possibility of autoimmune processes in COVID-19 myocarditis. Computational analyses have identified shared motifs between SARS-CoV-2 spike protein and human cardiac proteins [6,8]. A 2025 study in *Circulation* demonstrated that T cells from patients with vaccine-associated myocarditis recognized spike epitopes homologous to cardiac proteins and induced myocarditis in mice [9]. Anti-cardiac autoantibodies have been reported in 50–68% of severe COVID-19 patients and correlate with disease severity and outcomes [10,11].

Importantly, accumulating clinicopathologic data indicate that SARS-CoV-2-associated myocardial involvement is heterogeneous in presentation and pathology rather than a single uniform entity. Autopsy and biopsy studies have variably demonstrated viral material, inflammatory infiltrates, endothelial injury, and immune activation, with inconsistent detection of viral RNA within cardiomyocytes themselves [2,12,13,14]. In parallel, studies in hospitalized COVID-19 patients have described the emergence of new-onset and functionally active autoantibodies, reflecting systemic immune dysregulation with potential cardiac relevance [10,11]. Within this framework, functional immune assays evaluating antigen-specific cellular and humoral reactivity represent an additional approach for characterizing myocardial involvement beyond conventional histopathologic description.

Here we describe a patient with COVID-19 myocarditis in whom we evaluated cellular responses to SARS-CoV-2 spike protein and myocardial tissue and examined functional effects following passive transfer into immunodeficient mice.

## 2. Methods and Results

A 42-year-old man with a history of diabetes mellitus and hyperlipidemia presented to the intensive care unit with acute-onset chest pain and profound fatigue. Electrocardiography demonstrated diffuse ST-segment elevation with PR-segment depression. Laboratory evaluation revealed a peak troponin level of 17,104 pg/mL (reference 14 pg/mL), a white blood cell count of 12.6 K/µL, and a C-reactive protein (CRP) level of 15.4 mg/dL. Serologic testing indicated mildly symptomatic COVID-19 infection prior to admission. Transthoracic echocardiography demonstrated moderate left ventricular systolic dysfunction, while coronary angiography demonstrated non-obstructive coronary arteries excluding an acute coronary syndrome.

**Figure 1:**
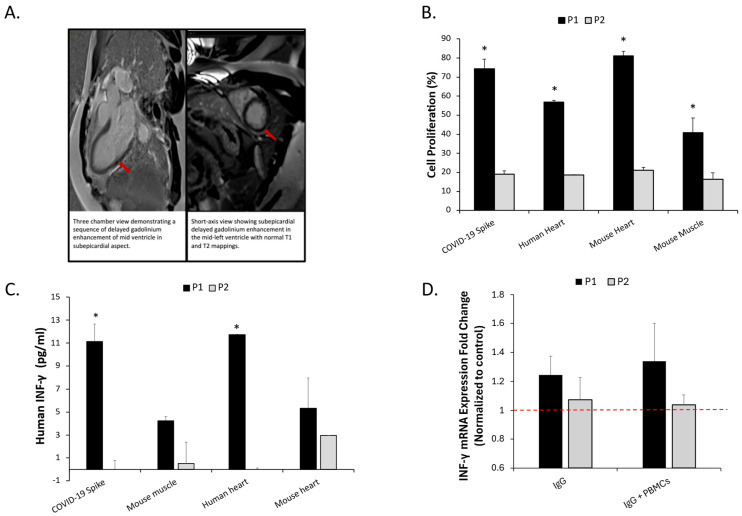
Integrated clinical and immunologic evidence of autoimmune myocardial injury in COVID-19. (**A**) Cardiac MRI demonstrating subepicardial late gadolinium enhancement and myocardial edema consistent with myocarditis (red arrows). (**B**) Proliferative responses of PBMCs from the myocarditis patient (Patient A = p1) and healthy control (Patient B = p2) following exposure to recombinant SARS-CoV-2 spike protein, human myocardial extract, or murine heart tissue. (**C**) IFN-γ concentrations in PBMC supernatants from Patient A and Patient B measured 24 h after exposure to spike protein or cardiac tissue extracts. (**D**) Cardiac IFN-γ mRNA levels in Rag2/IL2RG-deficient mice following administration of PBMCs or purified IgG from Patient A (P1) or Patient B (P2). The dashed red line indicates the normalized control level. * *p* < 0.05. P1: Patient A (Myocarditis patient), P2: Patient B (Healthy age-matched control).

Cardiac magnetic resonance imaging (CMR), performed with steady-state free precession cine sequences, T1 and T2 mapping, and late gadolinium enhancement (LGE), demonstrated focal subepicardial enhancement in the mid-ventricular lateral wall with associated myocardial edema (Figure 1A), fulfilling the updated Lake Louise criteria for myocarditis. Endomyocardial biopsy revealed mild hyperplastic changes with focal nuclear enlargement and minimal pericapillary lymphocytic infiltration of CD3 cells consistent with myocarditis. The patient was treated with anti-inflammatory therapy with clinical improvement and was discharged after 7 days with continued outpatient follow-up. PCR of myocardial tissue was negative for a panel of viruses including COVID-19.

Peripheral blood mononuclear cells (PBMCs) were isolated from the patient (Patient A) and from a healthy age-matched male without known chronic diseases (Patient B), with normal transthoracic echocardiography and no laboratory evidence of systemic inflammation at the time of blood sampling (Patient B). PBMCs from Patient A exhibited proliferative responses to recombinant SARS-CoV-2 spike protein (Figure 1B). To further determine whether cellular reactivity extended to cardiac tissue or other muscle-derived tissues, PBMCs were co-cultured with homogenized extracts of the patient’s myocardial biopsy, murine myocardial tissue, and murine skeletal muscle tissue (Figure 1B). These tissue extracts were included to assess whether the proliferative response was confined to myocardial tissue or reflected broader tissue cross-reactivity. PBMCs from Patient A demonstrated significantly higher proliferative responses compared with Patient B following exposure to spike protein, human myocardial extract, murine myocardial tissue, and murine skeletal muscle tissue (*p* < 0.05) (Figure 1B). Cell proliferation was quantified using a cell titer 96 assay (Promega, Madison, WI, USA).with absorbance measured at 570 nm, performed in technical triplicates.

To assess cytokine signaling consistent with T-cell activation, culture supernatants were collected and interferon-gamma (IFN-γ) concentrations were quantified by ELISA (R&D Systems, Minneapolis, MN, USA). Supernatants from Patient A showed markedly higher IFN-γ levels compared with Patient B (Figure 1C). IgG fractions were subsequently purified from the serum of Patients A and B using protein G chromatography. To evaluate whether human immune effectors could elicit myocardial immune activation in vivo, PBMCs or purified IgG from each patient were administered into Rag2/IL2RG-deficient (R2G2) mice (n = 15), a model lacking endogenous adaptive immunity and permitting human immune cell engraftment without graft-versus-host disease. No viral particles or infectious agents were introduced; only immune components were transferred. Upon sacrifice, murine hearts were dissected and processed for RNA isolation. Reverse transcription-PCR demonstrated increased cardiac IFN-γ transcript levels in mice receiving PBMCs or IgG from Patient A compared with those receiving PBMCs or IgG from Patient B (Figure 1D).

Human specimen collection was performed after written informed consent and approval by the Institutional Review Board of Kaplan Medical Center. Animal experiments were conducted under the oversight of the Institutional Animal Care and Use Committee of Kaplan Medical Center.

A summary of the experimental workflow and readouts is provided in Table 1.

## 3. Discussion

Myocardial injury and myocarditis have emerged as clinically relevant sequelae of SARS-CoV-2 infection, yet their underlying mechanisms remain incompletely defined. Beyond direct viral cytotoxicity, multiple reports have implicated immune-mediated pathways, including dysregulated innate responses, endothelial inflammation, microvascular injury, cytokine signaling, and molecular mimicry between viral and cardiac antigens [2,6,8,9,10,11]. Anti-cardiac autoantibodies have been detected in hospitalized COVID-19 patients and correlate with severity and outcomes, and computational analyses identifying shared motifs between the SARS-CoV-2 spike protein and human cardiac proteins further support the possibility of antigenic cross-reactivity [6,8,9,10,11]. In addition, autopsy series have shown that viral RNA is inconsistently detected in cardiomyocytes and is often localized to vascular or interstitial compartments, reinforcing the concept that immune-mediated injury likely contributes substantially in many patients [2,13,14].

Our findings extend this emerging framework to infection-associated myocarditis by demonstrating functional evidence of spike-directed immune responses with myocardial cross-reactivity and in vivo transferability. PBMCs derived from the affected patient exhibited proliferative responses to recombinant spike protein as well as to autologous myocardial tissue, accompanied by increased IFN-γ secretion—a cytokine central to T-cell-mediated autoimmune myocarditis and cardiomyocyte injury in experimental models [15,16,17,18]. Passive transfer of patient PBMCs or purified IgG into immunodeficient Rag2/IL2RG-deficient mice resulted in increased myocardial IFN-γ transcript levels, indicating that circulating immune mediators are capable of inducing myocardial inflammatory signaling in vivo. Taken together, the combination of spike reactivity, myocardial extract reactivity, and inducible cardiac inflammatory signaling supports the presence of functionally active, cross-reactive adaptive immune responses in this infection-associated case.

Beyond serving as a marker of T-cell activation, IFN-γ is mechanistically relevant in inflammatory myocarditis frameworks. Immune-mediated myocarditis is increasingly viewed as a dynamic process in which early Th1-dominant responses may shape downstream immune trajectories and myocardial remodeling patterns [19]. Experimental and translational models further indicate that cooperation between Th1- and Th17-associated immune programs is linked to progression from acute myocarditis toward inflammatory cardiomyopathy and a dilated phenotype in susceptible hosts [20]. Within this conceptual model, an early IFN-γ-dominant signature is consistent with acute immune activation, whereas interaction with additional adaptive pathways may influence chronic outcomes. Although our study did not include IL-17 profiling or longitudinal remodeling assessment, the demonstration of spike-reactive and myocardial cross-reactive immune activation with in vivo inflammatory signaling supports further longitudinal immune phenotyping to determine whether distinct immune response patterns are associated with persistent ventricular dysfunction [19,20].

Collectively, these observations support the concept that immune-mediated injury represents one contributory mechanism in COVID-19 myocarditis [2,10,11]. Importantly, this framework does not preclude additional parallel pathways, including endothelial dysfunction, microvascular thrombosis, and cytokine-driven myocardial injury [2]. The relative contribution of each mechanism likely varies across clinical phenotypes and may explain the broad spectrum of COVID-19 myocardial involvement ranging from subclinical myocardial injury to fulminant myocarditis [2,12].

A mechanistic study in vaccine-associated myocarditis demonstrated spike-reactive T-cell molecular mimicry with cardiac-homologous epitopes and myocarditis induction in mice, supporting biological plausibility for antigenic mimicry [9]. Our present study extends this concept to infection-associated disease and demonstrate functional immune transfer, thereby contributing additional context to the spectrum of spike-associated myocardial inflammation described in the literature. Differences in inflammatory context, antigen presentation, and host immune state may explain variability between infection-associated and other spike-associated myocarditis phenotypes.

The long-term cardiac consequences of SARS-CoV-2-associated myocardial involvement remain an area of active investigation. Systematic reviews and prospective convalescence studies have demonstrated that a subset of post-COVID patients exhibit persistent abnormalities in ventricular function and myocardial tissue characteristics, particularly among those with biochemical evidence of acute cardiac injury, and that residual myocardial scar is associated with increased adverse cardiovascular risk [21,22,23]. These observations underscore the importance of identifying mechanistic and immune determinants of incomplete recovery and support the need for immune-based phenotyping strategies that may help predict long-term outcomes.

Recognition of immune-mediated myocardial injury following SARS-CoV-2 infection is directly relevant to the interpretation of our findings. The observed spike-reactive cellular responses, myocardial cross-reactivity, and transferable inflammatory signaling are consistent with prior reports describing immune dysregulation and emergence of functional autoantibodies in COVID-19, as well as with established immune-mediated myocarditis frameworks [10,11,24]. Together, these data support the possibility that, in a subset of patients, myocardial injury may be driven in part by antigen-directed adaptive immune mechanisms rather than viral cytotoxicity alone. Within this context, immune profiling approaches—including assessment of antigen-specific cellular responses and cardiac-directed autoantibodies—represent a plausible strategy for improved mechanistic classification in future studies, particularly when integrated with clinical, imaging, and histopathologic data [10,11,24].

This study provides a functional immune framework for interpreting SARS-CoV-2-associated myocarditis and forms the basis for the clinical, methodological, and translational considerations outlined below.

## 4. Clinical Implications

Recognition of immune-mediated myocardial injury following SARS-CoV-2 infection is directly relevant in light of our functional findings of spike-reactive and myocardial cross-reactive adaptive immune responses with transferable inflammatory signaling. These results support the concept that a subset of patients may exhibit an immune-dominant mechanism of myocardial injury. Identification of immune signatures—such as antigen-specific T-cell responses, cytokine patterns, or cardiac-directed autoantibodies—may therefore assist in mechanistic phenotyping and in distinguishing immune-driven presentations from other forms of myocardial involvement. Such stratification may become clinically relevant when evaluating patients with suspected inflammatory myocarditis. The potential role of corticosteroids and other immunomodulatory strategies warrants further controlled investigation, particularly in biopsy-supported or severe inflammatory presentations, while maintaining caution in settings where ongoing viral activity has not been excluded.

## 5. Limitations

This proof-of-concept study is limited by its single-patient design and cannot determine the prevalence of immune-mediated mechanisms among patients with COVID-19 myocarditis. Immune characterization was restricted to proliferative assays and IFN-γ secretion without broader cytokine profiling or T-cell receptor sequencing. Although Rag2/IL2RG-deficient mice permit human PBMC engraftment without graft-versus-host disease, the model may not fully recapitulate the immunologic milieu of SARS-CoV-2 infection.

## 6. Future Directions

Future studies should evaluate whether spike-directed immune responses and myocardial cross-reactivity are present across larger cohorts of infection-associated myocarditis. Integrated immune profiling—including autoantibody characterization, T-cell receptor sequencing, and HLA typing—may identify pathogenic immune signatures and susceptibility patterns. Epitope mapping studies may clarify antigenic targets and inform antigen-specific tolerizing strategies. Longitudinal follow-up will determine whether immune-mediated injury contributes to persistent ventricular dysfunction in post-acute COVID-19 syndromes.

## 7. Conclusions

In this proof-of-concept study, infection-associated COVID-19 myocarditis was associated with spike-reactive adaptive immune responses that cross-reacted with myocardial tissue ex vivo and induced myocardial inflammatory signaling in vivo. These findings support a role for adaptive immunity in post-infectious myocardial injury following SARS-CoV-2 infection and justify further investigation into immune-mediated mechanisms in COVID-19 myocarditis.

## Figures and Tables

**Table 1:** Summary of immune assays performed.

Experimental Step	Material/Model	Input	Readout	Interpretation
PBMC proliferation	PBMCs (Patient A/B)	Spike protein/myocardial extract	Cell proliferation (absorbance 570 nm)	Evidence of antigen-specific cellular reactivity
IFN-γ secretion	PBMC supernatant	Spike protein/myocardial extract	ELISA	Evidence of T-cell activation
IgG isolation	Serum (Patient A/B)	Protein G chromatography	Purified IgG	Used for in vivo immune challenge
In vivo immune challenge	Rag2/IL2RG-deficient mice	PBMCs or IgG (Patient A/B)	Myocardial IFN-γ transcripts (RT-PCR)	Human immune effectors elicit myocardial immune activation in vivo

## Data Availability

The original contributions presented in this study are included in the article. Further inquiries can be directed to the corresponding authors.

## Abbreviations

ACE	angiotensin-converting enzyme
CMR	cardiac magnetic resonance
CRP	C-reactive protein
ECG	electrocardiogram/electrocardiography
ELISA	enzyme-linked immunosorbent assay
HLA	human leukocyte antigen
IFN-γ	interferon-gamma
IgG	immunoglobulin G
LGE	late gadolinium enhancement
LLC	Lake Louise criteria
LV	left ventricle/left ventricular
LVEF	left ventricular ejection fraction
mRNA	messenger RNA
OD570	optical density at 570 nm
PBMC	peripheral blood mononuclear cell
PCR	polymerase chain reaction
Prot-G	protein G chromatography
Rag2/IL2RG-/- mice	immunodeficient mice lacking adaptive immunity
R2G2	Rag2/IL2RG-deficient mouse model
RT-PCR	reverse transcription polymerase chain reaction
S protein	SARS-CoV-2 spike protein
SARS-CoV-2	severe acute respiratory syndrome coronavirus-2
ST	ST-segment
TCR	T-cell receptor
TTE	transthoracic echocardiography
WBC	white blood cell count
